# Reduced Prevalence of Malaria Infection in Children Living in Houses with Window Screening or Closed Eaves on Bioko Island, Equatorial Guinea

**DOI:** 10.1371/journal.pone.0080626

**Published:** 2013-11-13

**Authors:** John Bradley, Andrea M. Rehman, Christopher Schwabe, Daniel Vargas, Feliciano Monti, Camilo Ela, Matilde Riloha, Immo Kleinschmidt

**Affiliations:** 1 Tropical Epidemiology Group, London School of Hygiene and Tropical Medicine, London, United Kingdom; 2 Medical Care Development International, Silver Spring, Maryland, United States of America; 3 Medical Care Development International, Malabo, Equatorial Guinea; 4 Ministry of Health and Social Welfare, Malabo, Equatorial Guinea; Arizona State University, United States of America

## Abstract

**Background:**

Previous studies demonstrated that fewer mosquitoes enter houses which are screened or have closed eaves. There is little evidence about the effect on malaria infection in humans that changes in house construction may have. This study examines the impact of protective housing improvements on malaria infection on Bioko Island.

**Methodology/Principal Findings:**

Data from the annual malaria indicator surveys between 2009 and 2012 were used to assess trends in housing characteristics and their effect on RDT confirmed malaria infection in household members. Odds ratios were adjusted for socio-economic status of the household.22726 children between the ages of 2 and 14 years were tested for *P. falciparum*. Prevalence of infection in those living in houses with open eaves was 23.0% compared to 18.8% for those living in houses with closed eaves (OR = 0.81, 95% CI 0.67 - 0.98). The prevalence of infection for children in screened houses was 9.1% versus 20.1% for those living in unscreened houses (OR = 0.44, 95% CI 0.27 - 0.71). The proportion of houses with closed eaves increased from 66.0% in 2009 to 74.3% in 2012 (test for trend *p* = 0.01). The proportion of screened houses remained unchanged over time at 1.3%.

**Conclusion/Significance:**

As a malaria control intervention, house modification has the advantages that it is not affected by the growing threat of insecticide resistance; it protects all household members equally and at all times while indoors; and it offers protection against a number of vector borne diseases. The study provides evidence in support of efforts to regulate or encourage housing improvements which impede vector access into residences as part of an integrated vector control approach to complement existing measures which have been only partially successful in reducing malaria transmission in some parts of Bioko.

## Introduction

The high degree of endophagy of *many malaria vectors* [1] suggests that impeding their entry into houses could protect their occupants from infective mosquito bites. It has been argued that improvements in housing contributed to the elimination of malaria in many parts of the world, including Italy, the USA and England [2–4]. Preventing mosquitoes from entering houses has additional advantages such as protecting all household members equally and at all times whilst indoors and offering protection against other vector borne diseases through integrated vector control. Indoor residual spraying (IRS) and long-lasting insecticidal nets (LLIN) have contributed greatly to malaria control over the past decade, but with an estimated 660 000 deaths due to the disease in 2010 there is a clear need for additional interventions [5]. Due to the emergence and spread of insecticide resistance in malaria vector mosquitoes in Africa, interventions not relying on insecticide have an important role to play [6].

Perhaps the most obvious way of preventing mosquitoes from entering a house is to screen the entry points with a mesh. A trial of house screening in The Gambia showed that screening lead to a reduction in the number of mosquitoes entering houses and in the prevalence of anaemia in children, but did not show a reduction in malaria infection prevalence [7]. Other studies have shown that screening can be effective in preventing mosquito entry into houses [8] but there is little evidence as to how much screening can reduce malaria infection. Many houses in Africa have open eaves (i.e. a gap between the top of the wall and the roof), particularly in rural areas, and it has long been realised that this is an important point of entry for mosquitoes [9]. It has been demonstrated in many studies that closing the eaves reduces the number of mosquitoes entering a house [8,10–13], but again there is little evidence on the effect closing eaves would have on those living in such houses.

This study examines the effect of closed eaves and house screening on malaria infection on Bioko by examining data collected from malaria indicator surveys during the second 5 year phase of the Bioko Island Malaria Control Project.

## Methods

### Study Area

Bioko Island is part of Equatorial Guinea and lies 32 kilometres off the coast of Cameroon. The population of Bioko is approximately 250 000. Malaria is endemic, with continuous year round transmission. Before the launch of the Bioko Island Malaria Control Project (BIMCP), malaria transmission was high, with annual entomological inoculation rates (EIR) of over 750 and 250 infectious bites per person per year by *Anopheles funestus* and *An. gambiae* respectively [14], and a malaria prevalence of 45% in 2-14 year old children [15].

The BIMCP, funded by the Government of Equatorial Guinea and a consortium of private donors led by Marathon Oil Corporation, started an intensive malaria control strategy in 2004. The third five year phase of the project will begin in 2014. IRS was started in 2004, with a first round of deltamethrin followed by biannual rounds of the carbamate insecticide Bendiocarb (Ficam^TM^, Bayer) from 2005 to 2012. A satisfactory level of coverage has consistently been achieved [16,17]. In 2005, intermittent preventative treatment for pregnant women (IPTp), case management using artemisinin-based combination therapy (ACT), confirmation of diagnosis through microscopy and the introduction of rapid diagnostic tests (RDT) together with the training of health facility staff were introduced as additional measures. In 2007, 110 000 PermaNet 2.0 (Vestergaard Frandsen, Lausanne, Switzerland) LLINs were distributed to over 38 000 households through a mass distribution campaign. Initially very high levels of LLIN ownership and usage were achieved [15] but coverage has declined over time and in 2011 only 5% of 2 - 14 year old children were reported to be sleeping under a LLIN [17].

Malaria prevalence in 2-14 year old children dropped from 45% before the start of interventions to 32% in 2005 and 26% in 2006 [15]. After several years of little further change, prevalence declined to 14% in 2012 (see results section). Moderate to severe anaemia (Hg < 8 g/dL) fell from 15% to 1% over the life of the project, and all cause under-five mortality fell from 152 per 1,000 births to 55 per 1,000 in the first four years post intervention [15].

The discovery of large deposits of oil and gas in the 1990s has transformed Equatorial Guinea from a poor, mainly agricultural economy, into one of the fastest growing economies and foremost oil producers in Africa. Growth in real per capita Gross Domestic Product has averaged 4.3% per year since 2004, and by 2011 the country had the highest per capita Gross National Income in Africa at US$ 14,540 [18]. Throughout the last decade, the Government of Equatorial Guinea has invested extensively in the development of basic infrastructure including roads, schools, hospitals and social housing, though much of the latter remains to be inhabited. Coincident with this public sector infrastructure development has been a huge growth in private housing. Housing data enumerated by the BIMCP show that the number of houses on the Island has increased by approximately 20% per year since the inception of the project in both urban and rural areas, from an estimated 18,800 houses in early 2004 to 53,600 in 2012. Much of the new housing construction has been carried out in an unplanned manner with little evident adherence to building codes. 

**Figure 1:**
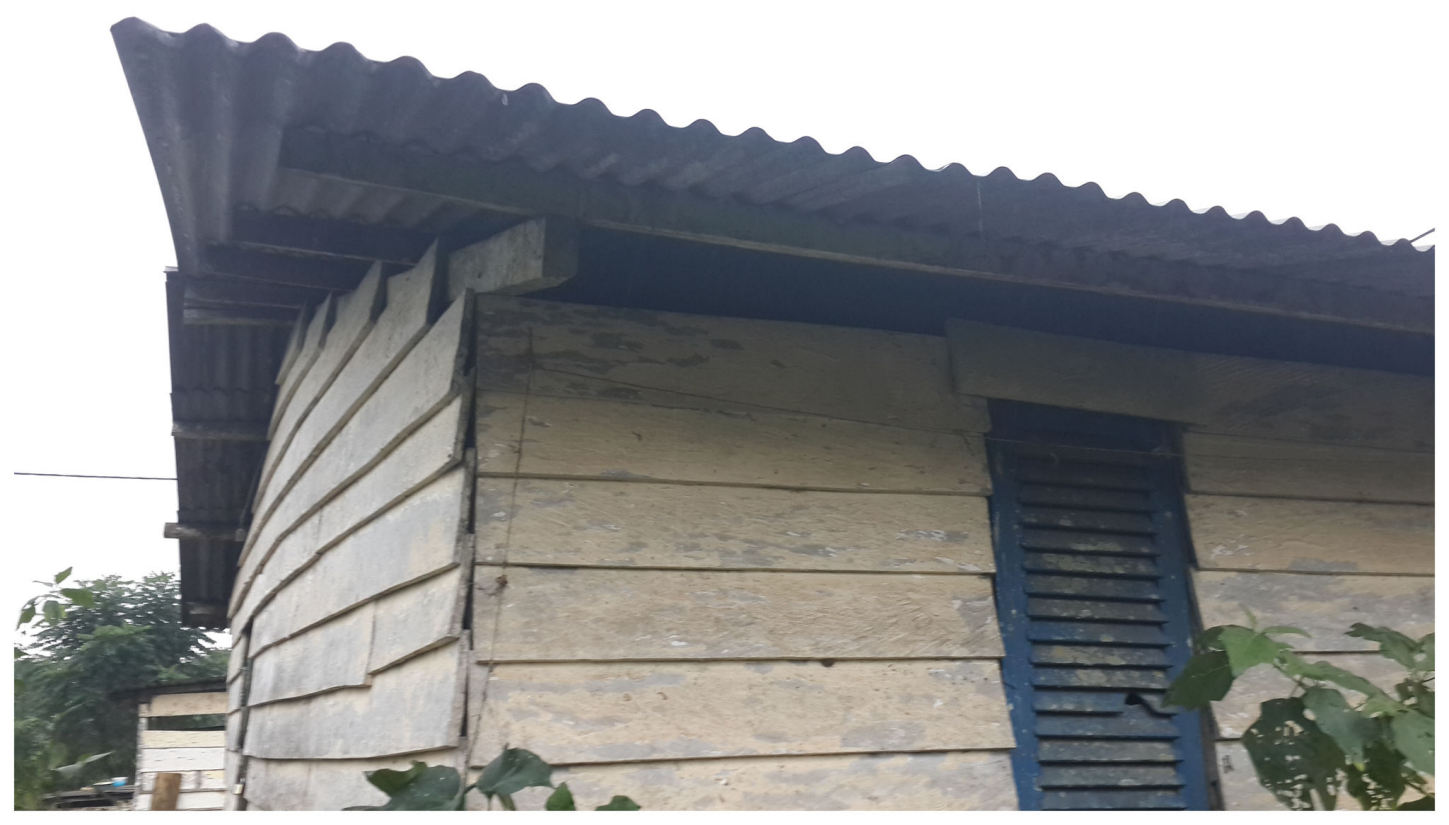
An example of a house on Bioko with wooden walls and open eaves.

Due to high levels of rainfall mud houses are very rare on Bioko. Nearly all houses (99%) have either wood or cement walls. Figure 1 shows a typical house with wooden walls and open eaves. In this study houses were classified as having wooden walls or not.

### Monitoring

Household malaria indicator surveys have taken place annually on Bioko since 2004 [15–17,19–21]. The data for this study were taken from the 4 surveys of the second phase of the project which took place in August and September of 2009, 2010, 2011 and 2012. Monitoring of the impact of the BIMCP is based on a system of eighteen sentinel sites, of which five are in Malabo (urban). In each survey, houses were sampled randomly at each site using lists constructed by enumerating all households at a site. Personal digital assistants (PDAs) equipped with global positioning systems (GPSs) were used to record data. The survey instrument was adapted from the malaria indicator survey developed by the Roll Back Malaria Monitoring and Evaluation Reference Group [22]. For each house it was assessed whether doors and windows were screened and whether there was glass in the windows; and recorded as none, some or all. A house was considered totally screened if all doors were screened and either all windows were screened or all windows had glass. A house was considered partially screened if at least one door or window was screened or at least one window had glass in. Sample size was determined to show a change in prevalence of infection from 20% to 17% between years, assuming a design effect of 2.5. Children 2- 14 years old had their haemoglobin measured (HemoCue, Ängelholm, Sweden) and were tested for *Plasmodium falciparum* using RDT (ICT™ Malaria Combo Cassette Test ML02, R&R, Cape Town, South Africa), subject to informed written consent from a caregiver. Children testing positive for parasitaemia, with haemoglobin <11 g/dL or who were febrile, were referred to a local field clinic for appropriate treatment (anti-malarial, anti-pyretic, or iron supplementation). In accordance with malaria indicator surveys in high endemicity settings [22], testing for malarial parasites was restricted to the child population since parasitaemia in adults may be modified by their immune response. 

### Statistical analysis

The average number of occupants per household, the average number of rooms, the average number of people per room, the proportion of houses with wooden walls, the proportion of houses with dirt floors, the proportion with closed eaves and the proportions in the 2 categories of screening were tabulated for each survey and overall. Either linear regression (in the case of number people and rooms), logistic regression (in the case proportions of wood walls, dirt floors and closed eaves), or ordered logistic regression, (in the case of categories screening), with year of survey as a linear independent variable was used to assess trends over time.

Household socio-economic status (SES) was calculated using the first principal component score based on variables related to household size, asset ownership, livestock ownership and household utilities. The SES scores for each survey year were converted to quintiles for analysis. Analysis of the association between household characteristics and SES was carried out in the same way as for time trends described above.

In 2008, filter paper blood samples were collected from 7387 individuals of all age groups, for detection of antibodies to *P. falciparum* Apical Membrane Antigen-1 (AMA-1) by enzyme linked immunosorbent assay (ELISA), for calculating sero-prevalence by age and for estimating the serological conversion rate (SCR) for each sentinel site [23]. SCR has been shown to be a reliable marker of the intensity of malaria transmission [24,25]. In this study, the previously estimated SCR of each sentinel site was used as a proxy to adjust for the site-specific, underlying transmission intensity.

Multivariable logistic regression was used to investigate the relationship between household characteristics and RDT confirmed *P.falciparum* infection in 2–14 year olds. Crude odds ratios were calculated, adjusted for survey year only. Adjusted odds ratios were calculated, adjusting for the following factors previously shown to be associated with malaria prevalence in Bioko [17]: whether the IRS spray coverage was over 70% at the site in the previous 6 months, individual net use, quartile of site specific SCR, age of child (grouped as 2-4, 5-7, 8-11 and 12-14 years old), household SES by quintile, and living in an urban or rural area. Household characteristics shown to have an association with malaria prevalence in the univariable analysis were also adjusted for. 

In all analyses standard errors were adjusted for the survey design by taking into account the between site variation in prevalence using the svy set of commands in Stata [26,27]. The primary sampling unit (PSU) was set to be the site. All analyses were performed using Stata version 12 [28].

### Ethics and informed consent

Ethics approval for the study was granted by the Equatorial Guinea Ministry of Health and Social Welfare and the ethics committee of the London School of Hygiene and Tropical Medicine (approval number 5556). Informed written consent was given by each participant or, in the case of children, a responsible adult. In the case of participants being unable to read, the text was read and explained to them, and consent was confirmed by an independent witness identified on the consent form.

## Results

The number of households surveyed in 2009, 2010, 2011 and 2012 respectively was 2245, 2938, 2819 and 2791. The proportion of houses with closed eaves increased over time from 66.0% to 76.4% (*p* = 0.01) but there was no evidence of a change in the proportion of houses screened (Table 1). The average number of rooms per household increased over the 4 survey years from 4.5 to 5.5 (*p*< 0.01) but the average size of the household did not, meaning that the average household occupancy decreased from 1.36 to 1.01 persons per room (*p*< 0.01). 

**Table 1:** Household characteristics during the 4 survey years.

	Survey				*p*-value of test for trend
	2009	2010	2011	2012	
Average number of:					
People per house	5.5	4.9	4.8	5.2	0.35
Rooms per house	4.5	4.8	5.0	5.5	<0.01
People per room	1.36	1.14	1.05	1.01	<0.01
Houses with, % (N):					
Wooden walls	59.9% (2230)	62.0% (2932)	56.7% (2811)	55.5% (2788)	0.38
Dirt floors	5.9% (2243)	5.6% (2938)	3.2% (2815)	3.4% (2790)	0.02
Closed Eaves	66.0% (2242)	63.8% (2938)	67.3% (2813)	74.3% (2790)	0.01
Partial screening	12.9% (2242)	11.7% (2938)	15.0% (2815)	13.2% (2790)	0.40
Total screening	1.3% (2242)	0.7% (2938)	0.9% (2815)	2.0% (2790)	

Urban and rural households had a similar average number of residents, 5.1 and 5.0 respectively. Rural houses had a slightly larger number of rooms on average (5.2 vs 4.9) but this difference was not significant (*p* = 0.12). Dirt floors were more common in rural sites (9.2% vs 1.6%, *p*< 0.01) as were wooden walls (65.0% vs 54.6%, *p* = 0.17). Houses in urban sites were more likely to have closed eaves (76.1% vs 54.1%, *p*<0.01). Screening was significantly more common in urban houses (*p* < 0.01)., both partial (16.8% vs 7.3%) and total (1.5% vs 0.8%).

All household characteristics showed a strong association with SES (Table 2). Closed eaves and screening were more common in households with higher SES (*p*< 0.01). Households with higher SES tended to have more rooms (p < 0.01), more inhabitants (p < 0.01) and fewer occupants per room (*p*< 0.01). Higher SES was associated with fewer wooden walls (*p*< 0.01) and dirt floors (*p*< 0.01). 

**Table 2:** Distribution of household characteristics by SES quintile.

	SES quintile					*p*-value of test for trend
	1 (lowest)	2	3	4	5 (highest)	
Average number of:						
People per house	4.5	4.8	5.0	5.2	5.7	<0.01
Rooms per house	4.2	4.5	4.9	5.2	6.2	<0.01
People per room	1.27	1.22	1.13	1.07	0.98	<0.01
Houses with, % (N):						
Wooden walls	70.1%, (1985)	73.8% (2000)	65.4% (2000)	54.0% (2002)	30.7% (2001)	<0.01
Dirt floors	15.2% (2005)	4.7% (2002)	2.2% (2002)	0.8% (2002)	0.2% (2001)	<0.01
Closed Eaves	41.0% (2005)	56.4% (2002)	68.9% (2002)	81.4% (2002)	91.7% (2001)	<0.01
Partial screening	2.8% (2005)	5.7% (2002)	9.8% (2002)	14.3% (2002)	30.2% (2001)	<0.01
Total screening	0.1% (2005)	0.5% (2002)	0.7% (2002)	1.1% (2002)	2.9% (2001)	

The number of 2-14 year olds tested for *P. falciparum* malaria infection in the 2009, 2010, 2011 and 2012 surveys was 5107, 6470, 5422 and 5727 respectively (data not tabulated). The prevalence of infection in each year was 20.4%, 25.5%, 20.2% and 13.7% respectively, with overall mean of 20.1%. The prevalence of malaria infection in 2-14 year olds was similar in rural and urban (19.1% vs 20.7%, *p* = 0.72). The numbers of children who were reported to have slept under untreated and treated nets the night before the survey were 3059 (13.5%) and 3177 (14.0%) respectively. The number of children who lived in houses that were reported to have been sprayed within the last 6 months was 15023 (66.1%).

The 287 children who lived in totally screened houses had a substantially lower prevalence of malaria infection than those who lived in unscreened houses (9.1% vs 20.1%, *p* < 0.01), but those in partially screened houses did not have lower levels of infection than those in unscreened houses (Table 3). Closed eaves were associated with lower levels of infection (18.8% vs 23.0%, *p* = 0.03). Both these effects remained after adjusting for confounders. Infection prevalence was associated with the density of people living in a house in the unadjusted analysis (*p* = 0.01) but after adjusting for confounders there was no evidence of an effect (*p* = 0.14). The proportion of houses with wooden walls was 58.5 %; of the remainder, all but 1% had cement walls. Open eaves were more common in wooden houses than in cement houses (43.6% versus 15.8%, *p* < 0.01). Neither dirt floors nor wooden walls were associated with malaria prevalence (*p* = 0.50 and *p* = 0.23 respectively).

**Table 3:** Association of household characteristics with malaria prevalence in 2 - 14 year olds.

Effect of	Level	Infection prevalence %, (n)	Odds ratio* (95% CI)	*p*-value	Adjusted odds ratio^†^ (95% CI)	*p*-value
Number of people in house	1-4	19.3 (5963)	1	0.22	1	0.48
	5-7	19.7 (11269)	1.04 (0.90,1.20)		0.98 (0.86,1.12)	
	8 or more	21.8 (5491)	1.21 (0.97,1.51)		1.09 (0.85,1.38)	
Number of rooms	1-3	22.7 (3809)	1	0.36	1	0.53
	4-5	20.5 (9289)	0.92 (0.81,1.04)		1.10 (0.99,1.23)	
	6 or more	18.8 (9628)	0.87 (0.70,1.07)		1.08 (0.88,1.33)	
Number of people per room	<1	18.2 (10561)	1	<0.01	1	0.14
	1-2	21.2 (10341)	1.18 (1.04,1.34)		1.09 (0.95,1.26)	
	>2	25.1 (1821)	1.43 (1.17,1.75)		1.22 (1.01,1.48)	
Wooden walls	Yes	21.4 (13123)	1	-	1	-
	No	18.5 (9550)	0.85 (0.65,1.12)	0.23	0.89 (0.69,1.16)	0.37
Dirt floors	Yes	23.8 (1035)	1	-	1	-
	No	20.0 (21689)	1.20 (0.69,2.07)	0.50	1.05 (0.73,1.52)	0.76
Closed eaves	Yes	18.8 (15542)	1	-	1	-
	No	23.0 (7182)	1.23 (1.02,1.49)	0.03	1.30 (1.13,1.48)	<0.01
Screening	None	20.1 (19366)	1	0.01	1	0.01
	Partial	21.1 (3071)	1.09 (0.93,1.27)		0.98 (0.84,1.15)	
	Complete	9.1 (287)	0.44 (0.27,0.71)		0.39 (0.23,0.69)	

* adjusted for year of survey ^†^ adjusted for year of survey, spray coverage, net use, SCR, age, SES, living in an urban area, number of people per room, eaves and screening.

## Discussion

This study shows that children living in houses with open eaves on Bioko Island were at a higher risk of malaria infection than those living in houses with closed eaves even after adjusting for the SES of the household. Many studies have shown that mosquitoes are more likely to enter a house with open eaves [8,10–13]; for instance *Njie et al* [13]found that in the Gambia 65% fewer *An. gambie* s.l. entered houses with closed eaves. There has, however, been little evidence on whether this reduction in mosquitoes entering translates into lower malaria infection in those sleeping in the house. A study [29] in the Ethiopian highlands in 1997 did show an association with malaria incidence in children living in houses with open eaves, but made no adjustment for SES. Living in better houses and hence dwellings with closed eaves is very likely to be associated with higher SES; raised risk of malaria has been shown to be associated with lower SES [30]. Therefore any observed associations between house construction and malaria is very likely to be confounded by SES, unless it is controlled for in the analysis. Our data confirm that SES is associated with closed eaves and previous studies on Bioko [17] have shown that SES is negatively associated with malaria infection .

Prevalence of malaria infection in children living in houses reported as fully screened was substantially lower than in those living in unscreened houses. There was, however, no evidence of a difference for those living in partially screened houses compared to unscreened houses. This suggests that screening must be complete, or nearly complete, to be effective. A randomised trial in the Gambia showed screening to be effective at preventing mosquitoes entering a house and in reducing the level of anaemia in children, but did not show a reduction in prevalence of infection [7]. There is anecdotal evidence that house screening contributed to a reduction in malaria transmission in Dar Es Salaam, Tanzania [31]. As with eaves though, there is little data on how house screening affects malaria. 

Although only 1.3% of the houses surveyed over the 4 years were fully screened, this was a large study involving over 10 000 houses and 20 000 children, of whom 287 lived in screened houses and showing strong statistical evidence for an effect, even after adjusting for important confounders. This effect was consistent across different years, with odds ratios of 0.59, 0.29, 0.49 and 0.42 for infection in houses with complete screening compared to no screening in the four survey years respectively. The odds ratios for association between open eaves and malaria infection were similarly consistent over the four survey years, being 1.33, 1.34, 1.23 and 1.25 respectively.

The emergence and spread of resistance in malaria vectors to all 4 classes of insecticides currently used for malaria vector control is recognised as a serious threat for the effectiveness of insecticide based vector control methods and hence for malaria control and elimination [6]. One of the potential advantages of restricting entry of mosquitoes into houses is that it is an intervention that does not rely on insecticides and is therefore unaffected by insecticide resistance. Another potential advantage of house improvements and screening is the equity with which it protects all members of the household at all times while indoors [32], unlike LLINs which primarily give protection to those with a net during sleeping hours only. Perhaps the greatest benefit to house modification would be the potential for integrated vector control, offering protection from other vector borne diseases as well as malaria. There is evidence that interventions which impede mosquito entry to houses could protect inhabitants from filariasis, Rift Valley Fever and O’Nyong Nyong [33]. Screening has been shown to have high levels of acceptability [34] which could make it an effective intervention in settings with malaria transmission, but where low levels of nuisance mosquitoes make the population disinclined to use bednets. It has been estimated that screening houses would cost 10$ per person in The Gambia [7] and the cost of screening the windows in a house in Dar Es Salaam has been estimated at 21$-30$ [35]. Therefore research would be needed into how cost-effective screening is before it can be promoted more widely as a public health intervention. Further research needs to assess the level of protection offered by damaged screening, how often screening would have to be replaced or repaired, and to what extent householders can be incentivised to carry out repairs.

The protective effect of closed eaves and window screening remained after adjusting for LLIN use and IRS coverage. This suggests that there may be a benefit to these interventions in addition to the standard malaria prevention tools.

It has been shown in previous studies that mosquitoes are more attracted to houses with high occupancy [8]. In this study, although there was a crude association between occupation density and malaria prevalence, the effect almost disappeared when adjusted for confounders. There was no evidence that wall type had any effect on malaria prevalence, even though IRS was the principal vector control intervention and has been shown to be affected by wall substrate [36]. Similarly, floor type showed no association with malaria infection.

A limitation of this study is that it uses observational data and houses were not randomised to the interventions. Although many observed confounding variables were adjusted for in this analysis there could still be residual confounding. In particular there could be aspects of socio-economic status not accounted for in the asset based score used in this analysis. There is a possibility of some mis-recording of data for house screening; a small number of non-randomly selected houses reported as screened were revisited and some had solid shutters over the windows which the inhabitants reported were kept permanently closed rather than a mesh over the windows. Although the effect of closed eaves and screening on mosquito abundance in houses is well known, it is a limitation of this study that no entomological data were available to corroborate the effect on risk of infection in humans. 

Table 2 shows a general trend towards improvement in housing on the island, with houses getting bigger, less crowded, with fewer dirt floors and fewer open eaves. In the long term such a trend could lead to a reduction in malaria transmission over time, and contribute to elimination as it is thought to have done in other countries [2]. The data presented here suggest that improved housing characteristics, including closed eaves and screening, contribute to malaria control on Bioko Island. Efforts to regulate or incentivise housing improvements which impede vector access into residences should be considered as part of an integrated vector control approach. Regulations governing the construction of new housing, and particularly social housing, should require that eaves be closed and windows and doors be screened. Homeowners should be encouraged through IEC campaigns and possibly through Government subsidies to comply with such regulations by retrofitting existing housing. 

The proportion of houses with closed eaves on Bioko is high and increasing, but the proportion with screening has remained very small. The rigorous analysis of data from four annual surveys presented in this study provides robust evidence of tangible protection against malarial infection resulting from relatively minor house improvements. The high transmission that persists in parts of the island even after nearly a decade of comprehensive interventions, and the additive protective contribution that housing improvements can make to reduce the prevalence of infection, suggest that strategies to regulate and encourage the adoption of protective housing improvements should be seriously considered for inclusion in the overall malaria control programme. As malaria control ultimately progresses towards elimination, socio-economic factors, including housing quality, will have an increasingly important role to play. Malaria control programmes should consider targeted housing improvements as a sustainable additional intervention to reduce transmission. To generate stronger evidence of the efficacy of this intervention for policy makers and funders generally, we would urge that randomised trials with epidemiological, entomological and costing outcomes be carried out as soon as possible.
